# Loss of the Integrin-Activating Transmembrane Protein Fam38A (Piezo1) Promotes a Switch to a Reduced Integrin-Dependent Mode of Cell Migration

**DOI:** 10.1371/journal.pone.0040346

**Published:** 2012-07-05

**Authors:** Brian J. McHugh, Amanda Murdoch, Christopher Haslett, Tariq Sethi

**Affiliations:** 1 Medical Research Council Centre for Inflammation Research, University of Edinburgh, Edinburgh, Midlothian, United Kingdom; 2 Departments of Respiratory Medicine and Allergy, Kings College London, London, United Kingdom; University of Kentucky College of Medicine, United States of America

## Abstract

Lung cancer is one of the most common fatal diseases in the developed world. The disease is rarely cured by currently available therapies, with an overall survival rate of ∼10%. Characterizing novel proteins that offer crucial insights into the processes of lung tumour invasion and metastasis may therefore provide much-needed prognostic markers, and influence therapeutic strategies. Aberrant function of the integrin family of heterodimeric cell surface receptors is a common theme in cancer - investigation into novel integrin activity regulators may offer crucial insights into the processes of tumour invasion and metastasis and may reveal insights into potential therapeutic targets. We previously described that depletion of the novel multi-transmembrane domain protein Fam38A, located at the endoplasmic reticulum (ER), inactivates endogenous beta1 integrin affinity, reducing cell adhesion. We now show that depletion of Fam38A, also now known as Piezo1, causes anchorage independence and a switch to a reduced integrin-dependent mode of cell migration/invasion, a novel phenotype for this integrin-regulating protein. Normal lung epithelial cells show increased rates of migration by 2D time-lapse microscopy and increased capacity to invade into matrigel, despite having decreased integrin affinity. We confirm greatly depleted Fam38A expression in small cell lung cancer (SCLC) lines where a form of reduced integrin-dependent migration, i.e. amoeboid migration, is a known phenotype. We propose that loss of Fam38A expression may cause increased cell migration and metastasis in lung tumours.

## Introduction

Lung cancer, the most common fatal cancer in the Western World, accounts for 6% of UK deaths. Small cell lung cancer (SCLC, ∼20% of all lung cancers) is an extremely aggressive form of the disease – although approximately 40% of patients show a complete initial response to chemotherapy but only 15% of patients have longer-term survival [1], [2]. Definition of new prognostic markers for SCLC would assist medical and patient decision making, highlight potential new therapeutic strategies and improve future research design.

The integrin heterodimeric adhesion complex plays a fundamental role in adhesion between cells and their surroundings. Alterations to integrin function and/or expression are a common theme in most cancers, and are known to promote tumour invasion and metastasis [3]. Integrins comprise of one alpha and one beta subunit, with 24 heterodimeric combinations known in humans. Importantly, integrin heterodimers allow bi-directional relaying of signals across the plasma membrane via changes in integrin affinity [4], [5]. Integrin affinity can in turn be modulated by cytoplasmic signalling pathways within the cell, termed “inside-out” signaling [6]. Changes to the function and/or expression of integrin heterodimers can promote anchorage independent growth, invasion and metastasis in cancer cells [3], [7]. However, although integrin cell surface expression levels are frequently linked to tumorigenesis, the relationship between integrin ligand affinity and tumorigenesis is less well studied.

Integrin-mediated ligand binding is intrinsically linked to cancer cell migration and invasion. In integrin-dependent (i.e. mesenchymal) cell migration, cells adopt a polarized spindle-shaped morphology, using traction achieved by integrin binding to the surrounding extra cellular matrix (ECM) for motility [8]. Several cytoplasmic signalling proteins have been shown to be crucial in this process, including Rac and Cdc42 [9]. Additionally, the proteolytic activity of secreted matrix metalloproteases 1, 2 and 9 degrade the surrounding extracellular matrix. However, studies in some tumour cells where surface integrins have been ablated have demonstrated that this does not affect their ability to migrate [8], [10]. In this integrin-independent mode of migration, named ‘amoeboid’, cells adopt a more ellipsoid shape and rely on actin cytoskeleton rearrangement to ‘push’ through the ECM [11]. Amoeboid cell migration has been shown as an alternative to both adhesion- and proteolytic-dependent mechanisms [11] – switching between mechanisms can be dependent on extracellular environment and internal molecular make-up. SCLC cells can utilise amoeboid movement during metastasis [10], linking integrin inactivation in these cells with their highly metastatic ability.

We previously identified the ER trans-membrane protein Fam38A as an activator of integrin affinity [12] that plays a key role in epithelial cell adhesion. The *Fam38A* chromosomal locus (16q24) is associated with loss of heterozygosity (LOH) in breast cancer, and is affected in some lung and gastric cancers [13], [14]. LOH in SCLC is more than 60% at numerous loci, including 3p, 5q, 11q, 13q, 17p and 22q [15] – at 22q13 LOH frequency surpasses 80% [16]. We therefore sought to address whether Fam38A expression was affected in the aggressive lung cancer SCLC, and whether loss of Fam38A expression would alter cell migration of normal lung epithelial cells.

## Results

### Fam38A Expression is Reduced in SCLC Cell Lines

**Figure 1:**
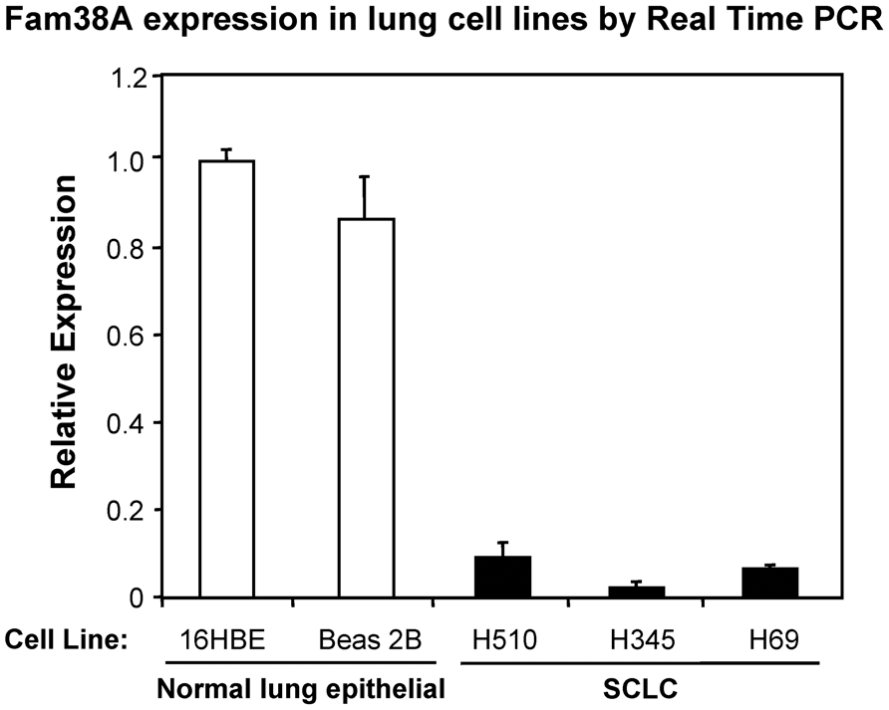
Fam38A expression is reduced in SCLC cell lines. Fam38A expression was measured by Real-time PCR in the indicated cell lines. Expression analysis showed a severe reduction (>90%) of Fam38A expression levels in SCLC cells, compared to 16HBE and Beas2B normal lung epithelial control cells.

Using Real-time PCR, we compared the level of Fam38A expression in the normal lung epithelial cell lines 16HBE and Beas2B to expression levels in the SCLC lines H345, H510 and H69. Figure 1 shows that SCLC lines had a striking reduction of expression compared to either 16HBE or Beas2B cells, ranging from approximately 10% in H510 to less than 2% in H345 cells. This result is intriguing, as SCLC is a particularly aggressive and metastatic form of lung cancer, and we have shown previously that integrin involvement in the establishment of anchorage independence and chemo-resistance in SCLC.

### Loss of Fam38A in Lung Epithelial Cells Reduces Integrin Affinity and Cell Adherence, and Leads to Increased Colony Formation in Soft Agar

**Figure 2:**
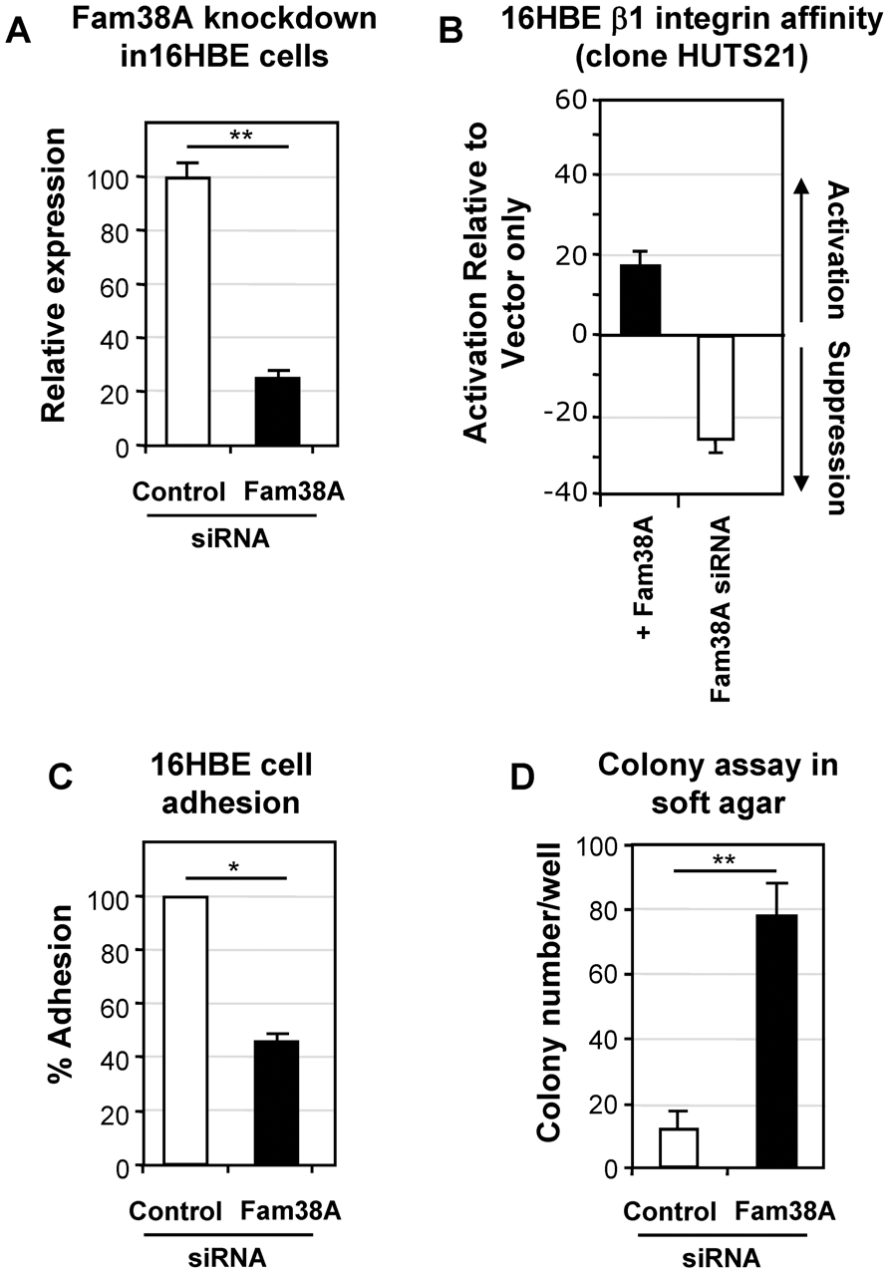
Fam38A siRNA causes cell detachment and anchorage independence in normal bronchial epithelial 16HBE cells. (**a**) Quantitation of Fam38A knock-down in 16HBE by Real-time PCR, compared to control siRNA treated cells. (**b**) Flow cytometry quantitation of control- and Fam38A-siRNA cells stained with the affinity-dependent integrin beta1 integrin antibody CD29 (HUTS-21). Fam38A-depleted cells show a reduction in HUTS-21 staining to 54% (+/−5%) that of control cell. N = 4. (**c**) Adhesion of control and Fam38A-siRNA cells - Fam38A-siRNA depleted cells showed a significant reduction in adhesion down to approximately 20% that of control cells. N = 3. (**d**) Colony formation in soft agar of 16HBE treated with control- or Fam38A-siRNA, showing a significant increase (approximately 7-fold) in colony number in Fam38A-depleted cells. N = 4.

We next sought to determine whether siRNA knockdown of Fam38A in normal lung 16HBE cells would replicate the reduced integrin affinity and adhesion phenotypes previously shown in HeLa cells [12]. Figure 2A demonstrates that Fam38A siRNA resulted in 76% knock-down in 16HBE cells, to 24% (+/−2%). A second oligo targeting the Fam38A gene was also shown to significantly reduce Fam38A expression to 31% (+/−3%) (data not shown). A corresponding decrease of beta1 integrin affinity in Fam38A siRNA-treated 16HBE cells was seen using the affinity-dependent beta1 integrin antibody CD29 (HUTS-21). Figure 2B shows a reduction in HUTS-21 staining in Fam38A-depleted cells to 54% (+/−5%) that of control cells. No significant difference in surface expression of beta1 integrin was observed, as tested by binding of an affinity-independent antibody CD29 (K20) (data not shown). We next tested whether Fam38A knock-down resulted in a corresponding lack of cell adhesion – Figure 2C shows that 16HBE cell adhesion was reduced to 43% (+/−3%), compared to treatment with a control oligo, although most detached cells were still viable, as measured by Annexin V staining (data not shown).

Anchorage independent growth is a central feature of cancer cells and is positively correlated with tumour aggressiveness [17]. Having established that reduced Fam38A expression in these lung epithelial lines phenocopied our HeLa cell data, we analyzed whether cell detachment led to anchorage independence in these lines. Cells were transfected with control- or Fam38A-siRNA and plated in soft agar to monitor colony formation. Figure 2D shows that Fam38A-depleted cells demonstrated a seven-fold increase in colony number after 2 weeks incubation, compared to control (Figure 2D), demonstrating that reduction of Fam38A leads to anchorage independent growth.

### siRNA Knock Down of Fam38A Causes Increased 2D and 3D Cell Migration

Cell migration in epithelial cells is commonly associated with higher levels of integrin expression. However, amoeboid migration has been described as a mechanism of metastasis for some tumour cells, including SCLC [8], [10], [11]. Based on its association with reduced integrin affinity, we hypothesized that reduction of Fam38A would bring about conditions skewed towards amoeboid migration in tumour cells. We therefore tested the effect of Fam38A siRNA on epithelial cell migration.

**Figure 3:**
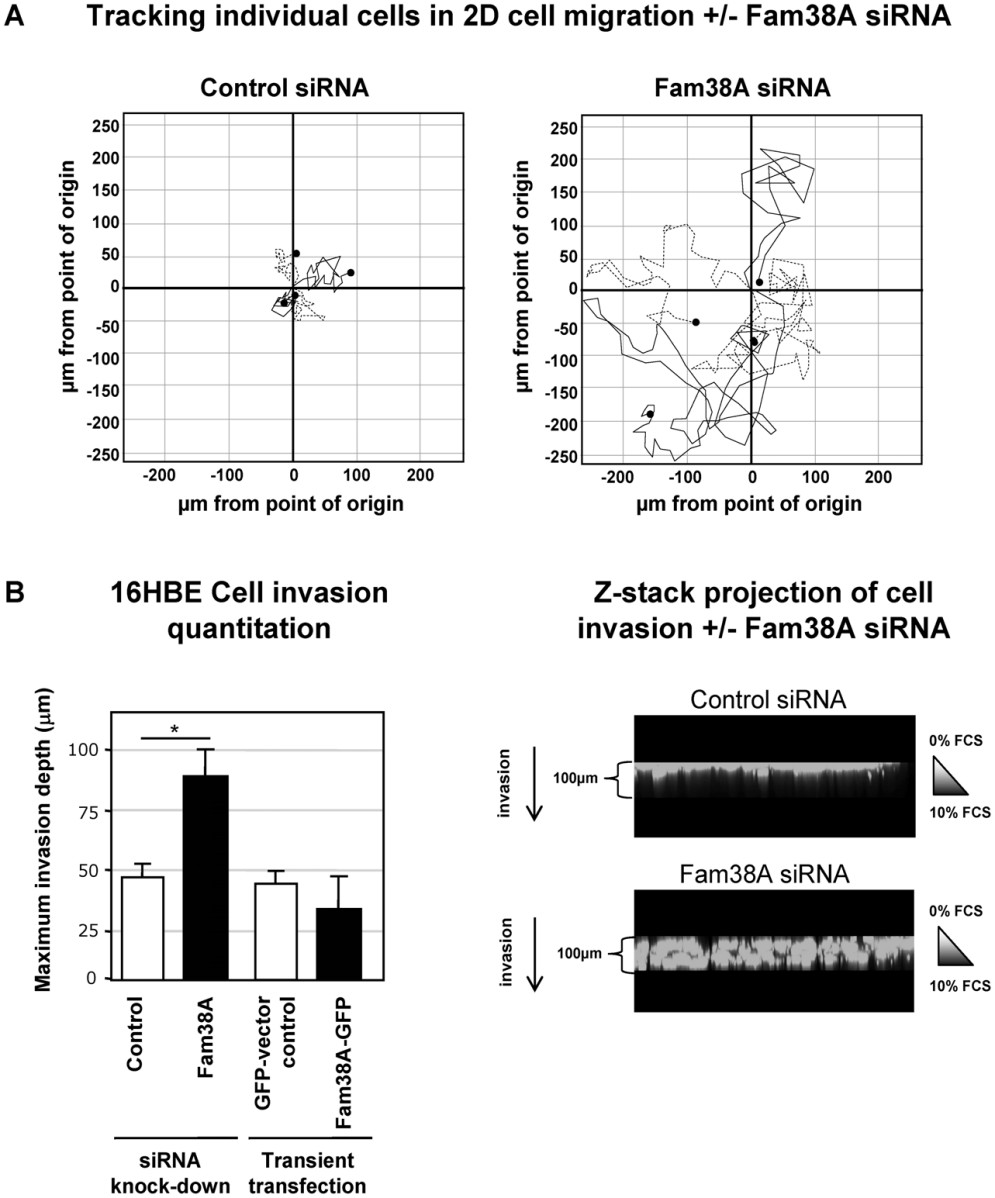
Fam38A-depletion results in increased 2D cell migration and 3D cell invasion intro matrigel. (**a**) Representative examples (4 per condition) of control- and Fam38A-siRNA treated 16HBE cell 2D migration using time-lapse microscopy. Fam38A-depleted cells migrate up to 250 µm from their starting point, in contrast to control cells which migrate up to 50 µm in the same time period. (**b**) Quantitation of invasion into matrigel of control vs. Fam38A-siRNA treated 16HBE cells, or GFP-vector vs. GFP-Fam38A transfected cells, towards a serum gradient. Representative confocal projections at a 90° angle to the stage are shown, demonstrating the extent of cell invasion.

Control oligo and Fam38A siRNA oligo-treated 16HBE cells were plated onto chamber slides and imaged by time-lapse microscopy for 8 hours. The migration of individual cells was then tracked over time by plotting the position of a migrating cell relative to its starting point (n = 45 individual cells per condition, over 3 separate experiments). Figure 3A shows 4 representative cells from each condition, demonstrating that isolated control cells migrated up to 50 µm on average from their starting point. However, Fam38A siRNA cells were seen to migrate further than their control counterparts, migrating on average up to 200 µm from their starting point (Figure 3A). In addition, Fam38A siRNA cells appeared to rapidly ruffle their membranes, and often had a period of rounding-up and detachment followed by re-attachment and cell spreading. These results demonstrate that, rather than simply detaching or failing to migrate after siRNA treatment, normal lung epithelial cells with Fam38A knock-down show increased cell migration compared to controls, despite having reduced beta1 integrin affinity.

These intriguing results led to us speculate that cells lacking Fam38A would also show increased cell invasion into extracellular matrix. We therefore tested invasion of control vs. Fam38A siRNA cells into matrigel, towards a serum gradient. We used a top-down approach, acquiring Z-stack images of 100 µm depth through the matrigel from where the initial layer of cells was plated - these z-stack images, in combination with 3D projection, were then used to quantitate the extent of cell invasion. Figure 3B shows that Fam38A-depleted cells invade through matrigel approximately twice as far as control cells. A representative projection of a z-stack series is also shown, to visualize the extent of cell invasion by these cells. As we previously showed that over-expression of Fam38A increases integrin affinity, we tested whether transient overexpression of a Fam38A-GFP clone would decrease invasion in 16HBE cells, in contrast to the Fam38A knock-down phenotype. Figure 3B shows that when GFP-labelled Fam38A was transiently over-expressed in 16HBE cells, these cells showed a tendency towards decreased invasion into matrigel, although not statistically significant in this experiment.

**Figure 4:**
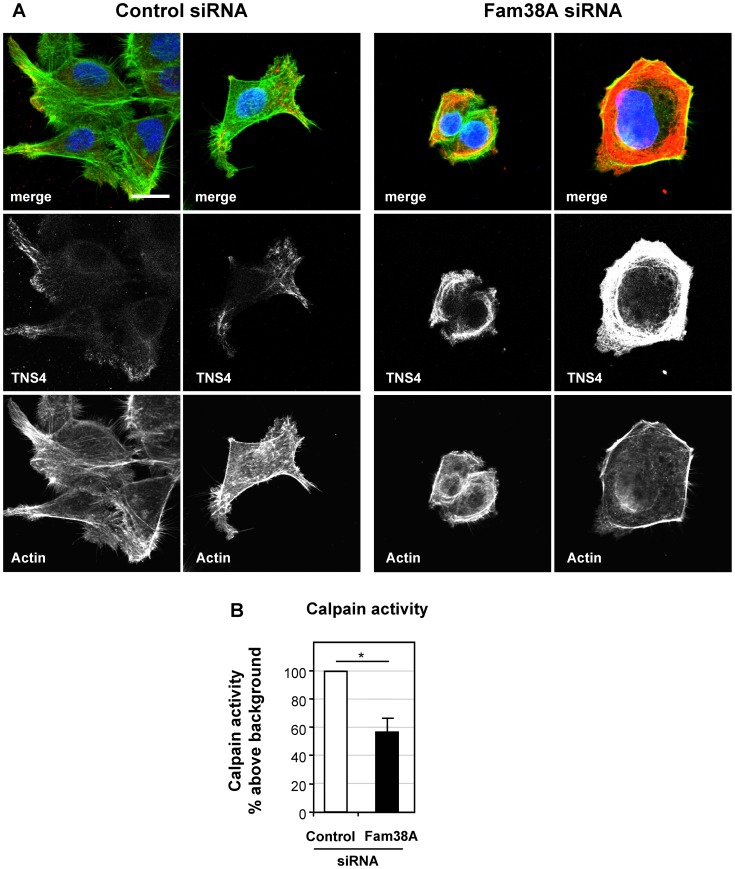
TNS4 upregulation, actin rearrangement and calpain activity in Fam38A-depleted cells. (**a**) Control- and Fam38A-siRNA treated 16HBE cells were stained for TNS4 (red channel), actin cytoskeleton (green channel), and nuclei (blue channel) showing upregulation of TNS4 expression and actin rearrangement in Fam38-depleted cells. Scale bar = 5 µm. (**b**) Quantitation of calpain activity (DABCYL cleavage) in 16HBE cells transfected with control- or Fam38A-siRNA. Mean N = 4, +/− SEM, p<0.05.

### Loss of Fam38A Expression Causes Upregulation of the Amoeboid Migration Marker TNS4, Actin Cytoskeleton Rearrangement and Reduced Calpain Activity

As we observed greater invasion in Fam38A-depleted cells compared to control (despite reduced levels of integrin affinity), we postulated that these cells may skew towards a form of amoeboid migration, which has been characterized in several tumour cell types, including SCLC. To test this we examined two phenotypic markers of amoeboid migration in these cells: actin cytoskeletal rearrangement and expression of the tensin family member TNS4 (also called cten), a known marker for amoeboid migration [18]. Control and Fam38A-siRNA cells were stained for TNS4 and phalloidin and examined by confocal microscopy. Figure 4 shows that individual Fam38A-depleted cells have higher levels of TNS4 staining than control cells. Additionally, Fam38A-depleted cells strikingly rearranged their actin cytoskeletal structure into a ring-like structure within the cytoplasm, rather than the actin fibres seen in control cells.

We next examined calpain activity in Fam38A-siRNA treated 16HBE cells. Calpain reduction is known to cause a switch to a reduced integrin-dependent invasion/migration pathway [19], which is known to occur in SCLC. We observed that calpain activity, monitored by quantitation of the fluorescent substrate “EDANS-EPLFAERK-DABCYL”, was reduced by 43% (+/−7%) in siRNA treated cells (Figure 4B). Based on these markers, and on the cell morphology and invasion phenotypes of Fam38A-depleted cells, we propose that these cells may be skewed towards an amoeboid form of migration.

## Discussion

We previously demonstrated that knock-down of the novel protein Fam38A results in beta1 integrin inactivation, a reduction in cytoplasmic calpain activity, and cell detachment. We now demonstrate that loss of Fam38A causes anchorage independence and increased cell migration/invasion in lung epithelial cells. We propose that Fam38A is likely to be a crucial candidate in creating a permissive phenotype for tumour cell metastasis, potentially via a form of ‘amoeboid’ migration.

Based on the size of Fam38A and its numerous predicted trans-membrane domains, we previously suggested it forms a membrane channel [12]. Our hypothesis was recently confirmed by a study on the mouse Fam38A homolog Piezo1, describing it as a mechanically activated cation channel [20]. This function may be distinct from its integrin activity-related phenotypes, as chelation of divalent cations with EGTA did not affect the ability of Piezo1 to act as mechanically activated channel [20]. Although we do not observe Fam38A at the plasma membrane in human cells in our studies, the possibility exists that relatively few Fam38A molecules are consistently present at the cell surface, with the majority residing (possibly transiently) at the ER. The recent association of mechanosensory function with Fam38A highlights the intriguing possibility that loss of Fam38A/Piezo1 may influence the ability of a cell to sense its environment through loss of a mechanosensory input, and contribute to cell rounding and ultimately to amoeboid migration.

Our results suggest that switching to a Fam38A-independent form of migration could cause a fundamentally important phenotypic change with drastic consequences for lung cancer cell metastasis. Fam38A could thus represent an important biomarker for increased metastasis in SCLC tumours.

## Materials and Methods

### Real-time PCR

Fam38A mRNA expression was monitored by Real-time PCR in the indicated cell lines, using a TaqMan pre-designed primer-probe (Applied Biosystems, Catalog no. Hs00207230_m1) and TaqMan Universal PCR Master Mix (Applied Biosystems). Analysis was performed using a 7900HT Sequence Detection System (Applied Biosystems), and SDS 2.1 software using the 2^−ΔΔCt^ method.

### Cell Culture and siRNA

16HBE cells (from D. Gruenert, University of California, San Francisco [21]) were maintained in DMEM supplemented with 10% (v/v) FBS, 1% L-glutamine, 1% penicillin/streptomycin, 1% non-essential amino acids. Serum free media without antibiotic was used for siRNA transfections. Fam38A siRNA oligo and transfection performed as described in [12].

### Flow Cytometry, Calpain Activity, Adhesion and Colony Assays

Staining of cells with the affinity-dependent beta1 integrin antibody CD29 (HUTS-21), and quantitation of calpain activity were performed as described in [12]. Adhesion of control and Fam38A-siRNA cells, were assessed by standard methods as described in [22]. Colony assays were performed as described in [23], 72 hours post-siRNA treatment, using 5×10^5^ treated live cells. Colonies were counted by phase microscopy after staining with nitro blue tetrazoleum solution.

### 2D Cell Migration Assays

Cells were plated into Chamber slides (Corning) at 37°C for 8 hours, and imaged at 5 minute intervals using a Leica DM IRBE microscope with motorized stage, Leica DFC280 camera and Leica QWin software. Cell migration was then analyzed using ImageJ (MTrackJ module) software.

### 3D Cell Migration Assays

Matrigel (BD Biosceinces**)** was diluted 1∶2 in serum-free media, and polymerized in 8 µm Transwell inserts for 1 hour at 37°C. Inserts were then placed in 24-well plates containing 10% FCS media. 5×10^4^ cells were diluted into 0% FCS media and layered directly onto the matrigel, and cells were allowed to invade for 48 hours at 37°C, then visualized (in non-GFP transfected experiments) by addition of 1 µM Calcein AM to the liquid media for 30 minutes at 37°C. Cells were imaged on a Leica TCS SP5 confocal microscope, with serial sections captured at 10 µm intervals.

### Immunofluorescence

Cells were prepared as described in [24], stained for TNS4 (mouse monoclonal antibody, Abnova), actin cytoskeleton (Alexa-488 phalloidin, Invitrogen), and nuclei (DAPI; Sigma-Aldrich), and imaged at room temperature on a Leica TCS SP5 confocal microscope, mounting medium Prolong Gold (Invitrogen). Images were captured using Leica Application Suite Advanced Fluorescence software, and adjusted (image cropping, brightness/contrast) in Adobe Photoshop.

### Statistical Analysis

Data were analyzed by one-way ANOVA and the appropriate post-test analyses were applied. p values <0.05 were considered to be significant.
